# Protocol-constrained AI enhances tacrolimus dosing accuracy in kidney transplant care

**DOI:** 10.3389/frai.2026.1825365

**Published:** 2026-05-21

**Authors:** Benjamin Bizer, Oscar A. Garcia Valencia, Flora Kincses, Jose Arriola-Montenegro, Charat Thongprayoon, Jing Miao, Wisit Cheungpasitporn

**Affiliations:** 1 Division of Nephrology and Hypertension, Department of Medicine, Mayo Clinic, Rochester, MN, United States; 2 Division of Pediatric Rheumatology, Department of Pediatrics, Mayo Clinic, Rochester, MN, United States

**Keywords:** artificial intelligence, clinical decision support, immunosuppressive therapy, kidney transplantation, large language models, tacrolimus

## Abstract

**Background:**

Tacrolimus dosing after kidney transplantation is complex, highly individualized, and prone to variability, which can impact graft outcomes. While machine learning (ML) approaches have been used primarily to predict tacrolimus concentrations, large language models (LLMs) may enable protocol-constrained clinical decision support by generating dosing recommendations aligned with established treatment guidelines.

**Methods:**

We developed TacroDose AI, a protocol-constrained LLM based on GPT-4, to generate tacrolimus dosing recommendations aligned with institutional guidelines. Using 300 structured simulated clinical scenarios, we evaluated protocol adherence, error patterns, and reproducibility of model-generated dosing recommendations. Following review of initial outputs, a refined prompt (*TacroAI 2.0*) was implemented to strengthen protocol constraints and structured output verification, and performance was reassessed.

**Results:**

TacroDose AI achieved 72.9% protocol adherence, with 27.1% protocol deviations, including 13.9% sentinel errors with potential clinical significance. Prompt refinement substantially improved performance: *TacroAI 2.0* achieved 91.7% adherence, reduced sentinel errors to 1.0%, and improved reproducibility from 77.3 to 86.7% (all *p* < 0.01). Although rounding discrepancies increased, these represented minor numerical differences without clinical relevance. Performance improvements were observed across subtherapeutic, therapeutic, and supratherapeutic tacrolimus scenarios.

**Conclusion:**

Protocol-constrained LLMs can produce guideline-concordant tacrolimus dosing recommendations with high adherence and improved reliability in simulated clinical settings. These findings highlight a potential framework for integrating generative AI with rule-based clinical protocols to support safe medication management. With further validation using real-world data, such systems could be integrated into electronic health records to support clinician-supervised, protocol-based immunosuppressive dosing in transplant care.

## 1 Introduction

Tacrolimus is the cornerstone of immunosuppressive therapy following kidney transplantation, and clinical guidelines recommend tacrolimus as first-line calcineurin inhibitor for maintenance immunosuppression [Kidney Disease: Improving Global Outcomes (KDIGO) Transplant Work Group, 2009]. Maintaining therapeutic tacrolimus serum concentration is critical, as both subtherapeutic and supratherapeutic drug levels can adversely affect renal allograft outcomes, increasing the risk of acute rejection or drug-related toxicity (Süsal and Döhler, 2019; Schumacher et al., 2021; Yang et al., 2025; Gaynor et al., 2016). Achieving this balance is challenging and influenced by multiple factors, including genetic polymorphisms affecting drug metabolism, drug–drug interactions, and patient-specific clinical factors in the immediate post-transplant period (Henkel et al., 2023). Tacrolimus intrapatient variability has been used as a metric to assess fluctuations in tacrolimus blood concentrations within individual patients over time (Shuker et al., 2015; Kuypers et al., 2026; Altynova et al., 2026; Francke et al., 2022). Higher intrapatient variability, reflecting less stable tacrolimus levels, has been associated with shorter graft survival (Javed et al., 2025; Lloberas et al., 2025).

This complex and dynamic environment frequently necessitates highly individualized dosing strategies. Despite the well-established role of tacrolimus in transplant immunosuppression, dose adjustments in routine clinical practice remain partly subjective and often vary across institutions and individual providers. This clinical challenge has motivated increasing interest in the use of artificial intelligence (AI)-based tools to support therapeutic drug monitoring and dosing decisions in transplantation (Cheungpasitporn et al., 2026; Tangri et al., 2025). This interest parallels a broader expansion of AI applications across nephrology, where emerging tools aim to support clinical decision-making in areas ranging from acute kidney injury prediction to dialysis and transplant management (Cheungpasitporn et al., 2026).

Several studies have explored machine learning (ML) approaches to predict tacrolimus pharmacokinetics and generate dose recommendations based on clinical and laboratory variables (Žilinská et al., 2016; Min et al., 2025; Yu et al., 2026). However, these models primarily function as pharmacokinetic prediction tools and rely on structured data inputs. The potential role of large language models (LLMs) as protocol-constrained clinical decision-support systems for tacrolimus dose adjustment has not been well explored.

In this proof-of-concept study, we developed *TacroDose AI*, a protocol-constrained model based on ChatGPT-4, designed to generate tacrolimus dosing recommendations aligned with the Mayo Clinic’s institutional tacrolimus dosing guidelines. Using simulated clinical scenarios, we evaluated whether a constrained LLM framework could produce guideline-concordant tacrolimus dosing recommendations, thereby exploring the feasibility of LLM-assisted decision support for tacrolimus dose adjustment.

## 2 Materials and methods

### 2.1 Study design

We conducted a two-phase simulation study to evaluate adherence of a GPT-based clinical decision support tool (*TacroDose AI*) to an institutional tacrolimus dose adjustment protocol. A total of 300 structured tacrolimus dosing scenarios were synthetically generated; no real patient data were used. Each case included three inputs: (1) most recent tacrolimus trough level (ng/mL), (2) current total daily dose (mg/day, combined AM/PM), and (3) target trough range (4–6, 6–8, 8–10, or 10–12 ng/mL). All cases assumed immediate-release tacrolimus administered twice daily, with identical input structure and formatting across simulations.

**Figure 1:**
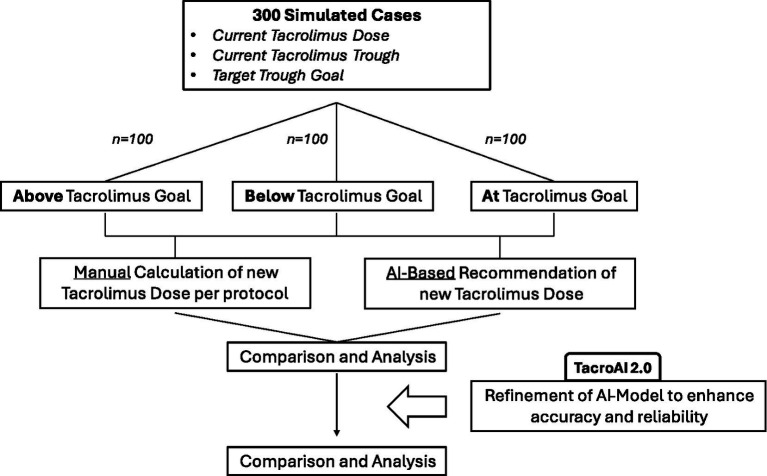
Two-phase simulation workflow for *TacroDose AI* (phase 1) and *TacroAI 2.0* (phase 2). Both AI models processed 300 structured tacrolimus dosing scenarios. Phase 2 used a refined, protocol-constrained prompt. Outputs were evaluated for protocol adherence, error types, and reproducibility across repeated scenarios.

The encoded protocol reflected institutional adjustment practices, consisting of predefined percentage-based dose modifications stratified by deviation from the target trough range, along with absolute supratherapeutic hold thresholds (see Figure 1).

### 2.2 Case construction

To reflect different clinical scenarios, trough levels were grouped by relation to the target range: 100 subtherapeutic, 100 therapeutic, and 100 supratherapeutic cases, with dose adjustments assigned according to protocol. Sub- and supratherapeutic cases were assigned specific percentage dose adjustments per protocol. For supratherapeutic levels exceeding hold thresholds, the protocol mandated holding one dose and rechecking the trough within 24–48 h without recalculating the maintenance dose. Trough values ranged from 2.5 to 15.4 ng/mL. Some clinical scenarios were intentionally repeated, appearing one to five times, to evaluate reproducibility of AI responses to identical inputs. Consequently, the dataset contained both unique and repeated scenarios, allowing assessment of both accuracy across distinct clinical situations and consistency of model outputs when identical cases were presented multiple times.

In addition, a small number of synthetically generated cases with target trough ranges outside the predefined institutional protocol (e.g., 10–12 ng/mL) were included for exploratory evaluation but were not part of the analysis, as no corresponding dosing guidance exists within the protocol framework.

The simulated clinical scenarios were constructed using structured inputs (e.g., laboratory values, dosing history, and predefined trough goals) to enable standardized evaluation of model performance. These scenarios did not incorporate unstructured clinical data or conflicting clinical signals. This controlled design was intentionally chosen to isolate and assess adherence to protocol-based dosing recommendations under consistent conditions.

### 2.3 Dose adjustment logic

The AI was instructed to follow institutional tacrolimus logic: apply all adjustments to the total daily dose (TDD), calculate percentage changes relative to the TDD, and round final doses to the nearest 0.5 mg. It was also directed to divide the TDD into twice-daily administration. For trough levels within the target range, no dose adjustment was recommended. Supratherapeutic hold thresholds were specified to override percentage-based adjustments.

### 2.4 AI models and prompt design

All cases were evaluated using ChatGPT 4.0 with a structured, protocol-constrained prompt incorporating the tacrolimus adjustment protocol.

#### 2.4.1 Phase 1: Baseline prompt

Each of the 300 simulated cases was processed individually using the initial structured prompt, which provided the model (TacroDose AI) with the full protocol and a fixed sequence of inputs. Outputs were recorded to identify inconsistencies or deviations from the protocol.

#### 2.4.2 Phase 2: Refined prompt

Following review of Phase 1 outputs, the prompt was revised to reinforce protocol adherence and structured output formatting (*TacroAI 2.0*). Modifications included stricter verification of total daily dose, cutoff thresholds, percentage-based calculations, and sequence of inputs. The same 300 cases were re-evaluated individually using the refined prompt to enable paired comparison of correctness and reproducibility.

### 2.5 Output review and error classification

All AI outputs were generated using a structured prompt requiring sequential reporting by the model of the current total daily dose, percentage adjustment, adjustment range (e.g., 10–20%), final total daily dose, and AM/PM split. This standardized format allowed each component of the recommendation to be systematically evaluated.

### 2.6 Protocol adherence

AI outputs were first classified according to protocol adherence, reflecting only aspects defined by the institutional tacrolimus dosing protocol:

- Protocol-adherent: was defined as outputs that correctly classified the dose adjustment (increase, decrease, or maintain), calculated the adjustment according to protocol, and produced a final total daily dose within the protocol-defined target range or within a clinically insignificant rounding margin of ±0.1 mg
- Protocol deviation: outputs that failed any of these criteria.

### 2.7 Error subclassification and hierarchical analysis

Protocol deviations were further categorized into high-level error categories to capture both clinical relevance and operational errors (Table 1):

1. Sentinel errors: deviations with potential for clinical harm, including directionally wrong adjustments, holding errors, excessive dosing (doubling/halving total dose or extreme percentage changes defined as exceeding twice the protocol-specified maximum), and categorization errors (incorrectly identifying whether the dose should be increased, decreased, or maintained).
2. Administration errors: deviations related to dose delivery, including improper splitting of the total daily dose between AM and PM doses.
3. Calculation/dosing errors: numeric deviations with limited impact, including minor percentage errors, rounding errors, or final doses slightly outside the expected range.
4. Missing output or formatting errors: absent, incomplete, or structurally non-conforming outputs, including failure to adhere to the predefined response format.

**Table 1:** Error classification framework used for AI output evaluation.

High-level category	Granular error examples
Sentinel errors	Holding error, directionally wrong adjustment, excessive dose change, categorization error
Administration errors	Incorrect splitting of total daily dose between AM and PM
Calculation/dosing errors	Percentage error, dosing range error, incorrect dose calculation, rounding errors
Missing output/formatting errors	Missing output, incomplete output, or deviation from the required structured response format

AI, artificial intelligence; AM, morning dose; PM, evening dose; TDD, total daily dose.

Within each category, granular error types were recorded to enable detailed performance analysis, allowing quantification of clinically significant and minor deviations.

A hierarchical classification approach was used for primary analysis, in which each output was assigned a single highest-severity error category (sentinel > calculation/dosing > administration > formatting) to avoid double counting downstream consequences of a single decision pathway. In addition to this primary classification, all individual error types were also recorded at the subcategory level, allowing outputs to contribute to multiple granular error counts.

Final total daily dose accuracy was analyzed separately as a component-level outcome to quantify the proportion of outputs resulting in an incorrect dose, regardless of upstream error type.

Rounding errors were defined as final recommended doses falling outside the calculated protocol-derived dose range, even when such deviations reflected standard tacrolimus dose increment constraints. In these cases, the model correctly identified the direction of dose adjustment, applied the appropriate percentage change, and generated the correct target dose range, but selected a final dose that fell outside this range due to rounding.

All outputs were independently reviewed by clinicians with expertise in renal transplantation, and discrepancies were resolved by consensus.

### 2.8 AI reproducibility assessment

To assess reproducibility, all repeated clinical scenarios were identified and the AI’s output for each instance recorded. Reproducibility was defined as the agreement rate: the frequency of the most common output divided by the number of repetitions. For example, if a scenario appeared three times with two identical outputs and one differing output, the agreement rate was 2/3 (~67%). Fully identical outputs were considered 100% reproducible, whereas completely discordant outputs had an agreement rate of 1/n, where *n* is the number of repetitions. The overall reproducibility rate was the mean agreement across all scenarios, reflecting how consistently the AI responded to identical inputs, independent of correctness.

### 2.9 Outcomes and statistical analysis

The primary outcome was protocol adherence, defined as exact concordance between model-generated recommendation and the encoded tacrolimus dosing protocol.

Secondary outcomes included total error rate, reproducibility and sentinel error frequency.

Because identical simulated cases were evaluated across both phases, paired analyses were performed. McNemar’s test was used to compare paired categorical adherence between Phase 1 and Phase 2. Paired t-tests were used to compare continuous deviation metrics. All tests were two-sided, and *p* < 0.05 was considered statistically significant.

## 3 Results

The performance of *TacroDose AI* was evaluated across 288 simulated tacrolimus dosing cases stratified by trough level relative to target range: 89 below-target, 100 at-target and 98 cases above target. Twelve cases were excluded by consensus review due to trough targets not specified within the institutional tacrolimus protocol. The remaining 288 cases constituted the final evaluation dataset and were used for both Phase 1 and Phase 2 analyses to enable paired comparisons.

### 3.1 Protocol adherence

**Figure 2:**
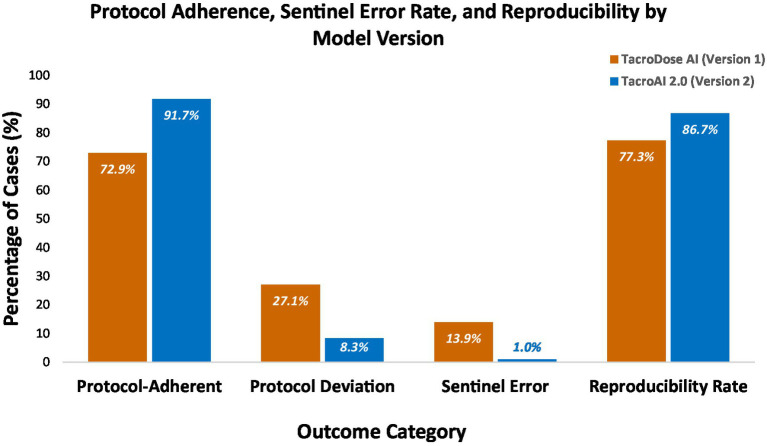
Protocol adherence, sentinel errors, and reproducibility by model version.

The initial GPT model demonstrated protocol adherence in 210 of 288 cases (72.9%), with deviations observed in 78 cases (27.1%). Following refinement, *TacroAI 2.0* showed a marked improvement, achieving adherence in 264 cases (91.7%) and protocol deviations in 24 cases (8.3%). McNemar’s test confirmed that the reduction in protocol deviations with *TacroAI 2.0* was statistically significant (*p* < 0.001) (see Figure 2).

Vertical bars show the percentage of cases for each outcome for the baseline GPT model (*TacroDose AI*) and the refined model (*TacroAI 2.0*). Protocol adherence and reproducibility reflect the proportion of cases meeting protocol criteria or producing identical outputs in repeated scenarios, respectively. Sentinel errors indicate potentially harmful deviations*. TacroAI 2.0* demonstrated substantial improvements: protocol adherence increased from 72.9% (210/288) to 91.7% (264/288), protocol deviations decreased from 27.1% (78/288) to 8.3% (24/288), sentinel errors dropped from 13.9% (40/288) to 1.0% (3/288), and reproducibility improved from 77.3 to 86.7%.

### 3.2 Error analysis

Major error categories, including sentinel, administration, calculation/dosing, and missing or formatting errors, were assessed for both models (Table 2). Compared with the baseline *TacroDose AI*, the refined model *TacroAI 2.0* demonstrated improvements across nearly all categories. Sentinel errors decreased markedly from 40 cases (13.9%) to 3 cases (1.0%), which was statistically significant by McNemar’s test (*p* < 0.0001). Errors involving incorrect final dose calculation decreased from 60 cases (20.8%) to 43 cases (14.9%). When considering all calculation-related error occurrences, including percentage errors, dosing range errors, incorrect dose calculations, and rounding discrepancies, the overall frequency of calculation/dosing errors also declined substantially (Table 2). Missing or formatting errors declined from 23 cases (8.0%) with the original GPT to 9 cases (3.1%) with *TacroAI 2.0*. Administration errors increased slightly from 1 case (0.3%) to 3 cases (1.0%), primarily due to incorrect splitting of AM and PM doses.

**Table 2:** *TacroDose AI* represents the initial implementation of the model, whereas *TacroAI 2.0* reflects the updated model with prompt refinements.

Error category	*TacroDose AI* (*n*)	*TacroDose AI* (%)	*TacroAI 2.0* (*n*)	*TacroAI 2.0* (%)	*p*-value
Major error categories					
Sentinel errors	40	13.9	3	1.0	*< 0.001*
Administration errors	1	0.3	3	1.0	*-*
Calculation/Dosing errors	225	78.1	112	38.9	*0.041*
Missing output/Formatting errors	23	8.0	9	3.1	*0.022*
Subcategories					
Holding error	11	3.8	2	0.7	*0.008*
Directional error	16	5.6	0	0	*< 0.001*
Categorization error	21	7.3	2	0.7	*< 0.001*
Excessive dose	20	6.9	0	0	*< 0.001*
Incorrect dose	75	26.0	23	8.0	*< 0.001*
Percentage error	40	13.9	7	2.4	*< 0.001*
Dosing range error	23	8.0	7	2.4	*0.006*
Rounding error	7	2.4	32	11.1	*< 0.001*
Missing/incorrect output	23	8.0	9	3.1	*0.022*
Administration error	1	0.3	3	1.0	*-*
Calculation error	60	20.8	43	14.9	*0.041*

*p*-values were calculated using McNemar’s test for paired categorical outcomes comparing Version 1.0 and Version 2.0 AI outputs. Major error categories represent the total number of error occurrences across all outputs and therefore may exceed the number of cases because multiple errors could occur within a single output. Percentages are calculated based on the total number of evaluated outputs per version. For subcategories with very low counts or zero occurrences, *p*-values may be unstable or are not reported.

The residual sentinel error rate (1.0%) of the *TacroAI 2.0* comprised three cases occurring near protocol-defined thresholds. Two involved above-target levels where the model did not recommend holding the dose and instead suggested reductions of 20–30% and 30–40%, respectively. One occurred in a below-target scenario, where the model correctly identified the need for dose escalation but recommended an increase exceeding twofold.

A more granular analysis of subcategories revealed further improvements. Directional errors, in which the model adjusted the dose in the opposite direction from protocol requirements, decreased from 16 cases in *TacroDose AI* to 0 cases with *TacroAI 2.0*. Categorization errors, where the AI incorrectly identified whether the dose should be maintained, increased, or decreased, declined from 21 to 2 cases. Considering the final recommended dose, incorrect dosing decreased from 75 cases to 23 cases. Rounding errors were more prominent in the refined model, increasing from 7 cases (2.4%) in *TacroDose AI* to 32 cases (11.1%) in *TacroAI 2.0*; this increase was statistically significant by McNemar’s test (*p* < 0.0001). Rounding discrepancies were more frequent in the subtherapeutic group (*n* = 26), with fewer cases in the supratherapeutic group (*n* = 6), and none observed within the therapeutic range. In the subtherapeutic group, 10 cases involved protocol-derived dose ranges (3.6–3.9 mg) that were not achievable with standard tacrolimus increments (0.5 mg or whole mg) and were classified as rounding errors despite no feasible in-range option. In the remaining cases, discrepancies were driven by selection of the upper bound of recommended dose increases (e.g., 30%) with rounding to the nearest available dose (e.g., 4.8 mg to 5 mg), resulting in doses slightly above the calculated range.

In the supratherapeutic group, all cases had feasible in-range dosing options; however, the model did not consistently select them. One case involved rounding below the target range, while the remainder reflected upward rounding beyond the upper limit.

Overall, discrepancies were driven by real-world dosing constraints and boundary selection within recommended adjustment ranges rather than calculation errors.

### 3.3 Error breakdown by trough level group

**Figure 3:**
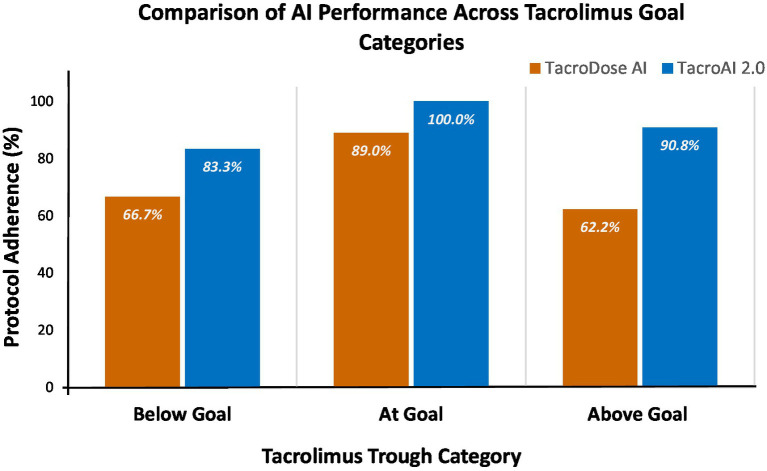
AI dosing adherence by goal category. Cases were grouped as below goal (*n* = 90), at goal (*n* = 100), or above goal (*n* = 98) based on initial tacrolimus levels. For each category, bars show the percentage of cases where each AI version adhered to target dosing, highlighting performance within each group. Differences between versions were assessed using the McNemar test.

Both AI versions performed worst in the below-target group, with the lowest protocol adherence. Rounding errors increased notably in this group in *TacroAI 2.0*, while dose and sentinel errors decreased. Notably, in the at-goal group, *TacroAI 2.0* eliminated all errors, including sentinel errors, achieving full reproducibility and highlighting the improved consistency of dose recommendations compared with the previous version. In the above-target group, overall errors declined substantially, driven by reductions in dose and sentinel mistakes. Adherence improved significantly across all tacrolimus goal categories with the improved AI model. In the below-target group (*n* = 90), *TacroAI 2.0* achieved significant improvements in adherence compared with TacroDose AI (*p* = 0.012). In the at-target group (*n* = 100), improvements were also statistically significant (*p* = 0.004), with complete elimination of errors in the refined model. In the above-target group (*n* = 98), *TacroAI 2.0* achieved adherence in 89 cases (90.8%), with a significant improvement compared with the baseline model (*p* < 0.001). McNemar testing demonstrated statistically significant improvements across all categories (Figure 3).

### 3.4 Reproducibility of AI responses

Reproducibility of AI responses was assessed using 211 repeated identical cases. For *TacroDose AI*, 163 of 211 repeated cases (77.3%) produced identical outputs. The refined *TacroAI 2.0* showed improved reproducibility, with 183 of 211 repeated cases (86.7%) yielding identical outputs. This improvement demonstrates enhanced consistency in prompt output with *TacroAI 2.0* compared to the baseline model and was statistically significant (McNemar’s test *p* = 0.002).

## 4 Discussion

In this study, we evaluated a protocol-constrained LLM to assess the feasibility of AI assisting with tacrolimus dosing in simulated kidney transplant cases. We observed a significant improvement in model performance after refining the prompt used to guide the LLM. Key modifications included providing stricter instructions to avoid deviation from the protocol, enforcing a more structured input–output sequence, and requiring verification of the current dose prior to generating recommendations. Following these adjustments, protocol adherence improved substantially, increasing from 73% to nearly 92%. Importantly, the rate of sentinel errors decreased to 1% in the updated *TacroAI 2.0* model. Among simulated cases in which tacrolimus levels were already within the target range, *TacroAI 2.0* achieved 100% adherence to the protocol.

The *TacroAI 2.0* produced a higher number of rounding discrepancies compared with the initial version, although these represented minor numerical differences without clinical relevance. This likely reflects increased protocol fidelity, with the model more consistently generating precise dose calculations that then required conversion to discrete, clinically available tacrolimus dose increments. In some cases, calculated dose ranges fell between standard dosing strengths, necessitating rounding at the final implementation step. Importantly, these discrepancies occurred alongside a substantial reduction in sentinel errors and major protocol deviations, suggesting an overall improvement in the safety profile of the model despite these minor inconsistencies.

Another important aspect of evaluating AI models is reproducibility, the ability of the model to generate consistent outputs when presented with the same input. For any potential clinical applications, lack of reproducibility could have significant negative consequences. The updated *TacroAI 2.0* demonstrated a substantial improvement in reproducibility following prompt modifications. Nevertheless, before implementation in patient care, outputs would need to be perfectly consistent to ensure safety and reliability.

In prior work, we evaluated AI-assisted erythropoiesis-stimulating agent (ESA) and iron management in dialysis patients using a rule-based, deterministic framework and observed near-complete protocol adherence (Arriola-Montenegro et al., 2025). In contrast, the tacrolimus model is based on a probabilistic LLM, which introduces inherent variability in output generation despite structured prompting and constraints. Accordingly, direct comparison of adherence rates between these systems should be interpreted cautiously given their different architectures. In the tacrolimus model, even with significant improvements in *TacroAI 2.0*, perfect concordance with protocol-specified dosing was not achieved. This likely reflects the greater complexity of tacrolimus adjustment, which requires integration of multiple clinical variables and small incremental dose modifications where minor interpretive or rounding differences can lead to deviations from exact protocol outputs. Despite this, the marked reduction in sentinel errors following prompt refinement supports the utility of protocol-constrained LLMs for clinical decision support in transplant care.

Previous studies have explored the application of AI in tacrolimus dosing: Huo et al. (2025) developed a long short-term memory (LSTM) model that, using medications, demographics, transplant timing, and other factors, predicted next-day tacrolimus concentrations with a mean absolute error of 1.88 ng/mL. In another study, models using only prior tacrolimus trough levels, dose, and route were sufficient to predict future doses (Choshi et al., 2025).

While these approaches address a critical aspect of transplant care and may reduce repeated laboratory measurements, most utilize ML and focus on predicting tacrolimus levels rather than providing individualized, guideline-based dosing recommendations.

By contrast, LLMs are primarily trained on unstructured text but can integrate both, unstructured and structured data. This enables the integration of narrative clinical context and guideline-based reasoning into dosing decisions. Unlike conventional ML models, which focus on predicting tacrolimus levels from numerical inputs, LLMs can generate protocol-concordant recommendations that directly support clinician decision-making. This approach represents a novel strategy for assisting with complex, individualized tacrolimus dosing, bridging the gap between AI-based prediction and actionable, guideline-aligned clinical guidance.

This study has several limitations. First, the analysis was conducted in a simulated framework and did not incorporate real patient data. As a result, the model’s recommendations could not be evaluated with respect to whether the proposed dose adjustments would have achieved target tacrolimus trough levels in clinical practice. However, the primary aim of this study was to evaluate protocol adherence and reliability of model-generated dosing recommendations rather than clinical pharmacokinetic outcomes. Second, the model was evaluated using a single institutional dosing protocol, which may limit generalizability to transplant centers with different tacrolimus adjustment strategies. In addition, the analysis did not assess the cumulative impact of sequential dose adjustments over time, leaving the longitudinal performance of the system untested. Finally, a further limitation relates to the structured nature of the simulated inputs. To enable controlled evaluation of protocol adherence, the scenarios did not include unstructured clinical data or conflicting signals (such as discordant laboratory trends, qualitative clinical observations, concomitant medications, intercurrent illness, suspected nonadherence, or pharmacogenomic factors) that frequently shape tacrolimus dose adjustments in routine practice. As a result, the present findings demonstrate that a protocol-constrained LLM can reliably reproduce a deterministic institutional dosing protocol, but they do not establish how such a model would perform when faced with the ambiguity, heterogeneity, and competing priorities of real-world transplant care. We have initiated follow-up work using de-identified real-world patient data that incorporates these contextual variables, longitudinal trough trajectories, and clinically discordant inputs, in order to evaluate model robustness, error patterns, and safety under conditions that more closely approximate clinical practice.

Future work should incorporate additional clinical variables, including responses to prior dose adjustments, concomitant medications, and pharmacogenomic factors known to influence tacrolimus metabolism. Prospective validation using real-world clinical data will be necessary to determine the clinical utility, safety, and reliability of such systems in routine transplant care (Kuypers et al., 2026; Rabindranath et al., 2024). In addition, future studies should evaluate the longitudinal performance of protocol-constrained models across sequential dose adjustments and assess their integration within electronic health record workflows to enable automated yet clinician-supervised dosing recommendations. Broader validation across multiple transplant centers with varying dosing protocols will also be important to determine generalizability and to establish whether such systems can support standardized, protocol-based immunosuppressive management in diverse clinical settings.

This study has several notable strengths. It represents one of the first applications of a LLM as a protocol-constrained decision-support tool for tacrolimus dosing. Model outputs were benchmarked directly against an established clinical protocol, providing clear, quantitative measures of adherence. Our results demonstrate that prompt refinement can substantially improve LLM performance, highlighting a practical approach to optimizing model recommendations. While this study is limited to simulated tacrolimus dosing in transplant care, it suggests a broader conceptual framework for protocol-constrained, individualized dosing systems that may be explored in other clinical settings in future research.

As a potential future clinical application, embedding this protocol-constrained model within the electronic medical record could generate tacrolimus dose recommendations that incorporate patient-specific clinical data and institutional dosing guidelines, while allowing clinicians to rapidly review and approve the suggested dose. This approach may help standardize and streamline a common, time-intensive, but crucial task in transplant nephrology.

## 5 Conclusion

This proof-of-concept study demonstrates that a protocol-constrained LLM can generate tacrolimus dosing recommendations with high adherence to institutional guidelines and improved reproducibility. Prompt refinement substantially reduced clinically significant errors, underscoring the value of structured guidance in LLM-based decision support.

Unlike conventional ML models that primarily predict drug levels, LLMs can integrate both structured and narrative clinical information, enabling guideline-concordant, individualized dosing. While evaluated in a simulated setting, this framework lays the groundwork for real-world implementation. Future studies should validate the model with patient data, incorporate additional clinical and pharmacogenomic variables, and assess longitudinal performance. Embedding such LLMs within electronic medical records could streamline tacrolimus management, reduce variability, and support safe, efficient, individualized immunosuppressive care.

## Data Availability

The original contributions presented in the study are included in the article/Supplementary material, further inquiries can be directed to the corresponding author.
